# Implementation of safety-related statutory information for herbal products under the pharmaceutical legislation between 1965 and 2021 in Finland: a content analysis of product information

**DOI:** 10.1186/s13690-025-01549-9

**Published:** 2025-03-05

**Authors:** Sari M. Koski, Anna Hietikko, Marja Airaksinen, Pirjo Laitinen-Parkkonen

**Affiliations:** 1 Faculty of Pharmacy, Division of Pharmacology and Pharmacotherapy, University of Helsinki, Viikinkaari 5 E, FI-00014 Finland; 2 Wellbeing services county of Central Uusimaa, Suutarinkatu 2, Hyvinkää, FI-05900 Finland; 3 Faculty of Medicine, University of Helsinki, Haartmaninkatu 8, FI-00014 Finland

**Keywords:** Herbal medicinal product, Medicines safety information, European Union, Finland, Pharmaceutical legislation, Implementation

## Abstract

**Background:**

The study aim was to investigate implementation of safety-related statutory information for herbal products under the pharmaceutical legislation between 1965 and 2021 in Finland. The study period covered Finland’s whole marketing authorisation system divided into five periods according to changes in legislation (1965–1982, 1983–1987, 1988–1993, 1994–2004 and 2005–2021).

**Methods:**

The study was carried out using qualitative content analysis focusing on herbal products granted marketing authorisations, registrations and licenses under the pharmaceutical legislation. Safety-related information in the summaries of product characteristics (SmPC), package leaflets (PL) and their predecessors (i.e., preliminary PLs called brochures) from 1965 to 2021 were analysed, particularly focusing on contraindications – including special warnings and precautions. The herbal product information data were searched from the archives of the Finnish Medicines Agency (Fimea). All searched information was from publicly available EU and Finnish legislations, administrative regulations, and approved product information of SmPC, PL and their predecessors.

**Results:**

The study material consisted of 207 herbal medicinal products after excluding six products because no data was found for them. Contraindications (including special warnings and precautions) were included in product information more systematically since 2005. Within 1965–1982, only 2/13 (15%) licensed products were equipped with a brochure. During 1994–2004 of the products 64/78 (82%) were equipped with the SmPC, PL or brochure. This legislation period can be seen as a watershed in implementation of SmPC into practice in Finland before the full (100%) implementation during the period 2005–2021. Of the contraindication (including special warnings and precautions) information, hypersensitivity/allergy information was included clearly most frequently.

**Conclusions:**

This study is based on a unique archive material covering years 1965–2021 in Finland. It shows successful national implementation of statutory product information for herbal products and how safety-related product information on contraindications (including special warnings and precautions) has improved during the study period, particularly since legislative changes in 2005. Currently, all herbal products under pharmaceutical supervision are medicinal products which are controlled like any other medicinal products for patient safety.

**Table:** 

Text box 1. Contributions to the literature
• Before this study, no research had been published on implementation of statutory herbal product safety information covering the whole period of the national authorisation system.
• This study provides a unique overview of the evolution of product information with selected safety-related topics in herbal products regulated under the pharmaceutical legislation in Finland.
• This study shows how harmonisation steps regarding herbal products regulated under the pharmaceutical legislation have been considered in an individual European Union member state and how this harmonisation has influenced the required and actually implemented safety information to improve public health.

## Background

Herbal medicinal products (HMPs) with active substances of vegetable origin have been part of the European pharmaceutical legislation since the first Pharmaceutical Directive 65/65/EEC came into force in 1965 [1]. The same directive in 1965 gave the first package leaflet (PL) requirements for the statutory consumer information [1] although the first official definition of herbal medicinal product was introduced as late as in 2004 in Directive 2004/24/EC [2]. The summary of product characteristics (SmPC) was introduced into the European pharmaceutical legislation in 1983 in Directive 83/570/EEC [3] to provide statutory product-specific medicines information to healthcare professionals. Directive 92/27/EEC [4], adopted in 1992, stated that the product information in PLs must be based on the information presented in SmPCs.

Finland became a member of EU in 1995. Some information requirements for product labelling and brochures intended for consumers, so-called preliminary PLs, were already given in Finnish pharmaceutical legislation in 1935 [5]. The first EU requirements for SmPC and PL were introduced to the Finnish national legislation in 2002 [6]. Instructions to add a statutory SmPC to the medicinal products that were already on the market, to new medicinal products, and to herbal remedies, were provided in the administrative regulations [7, 8]. These requirements have become more detailed over the years and are currently an important part of the EU’s pharmaceutical policy for patient safety. Today, the safety-related legislative requirements are practically identical for all medicinal products [9]. In this study, pharmaceutical safety-related requirement is defined as a parameter which is not an actual safety parameter, such as a safety test or a clinical trial but a parameter that has an indirect product-specific effect on the safety of a single herbal medicinal product.

The current study aimed to investigate implementation of safety-related statutory information for herbal products under the pharmaceutical legislations between 1965 and 2021 in Finland. The study period covered Finland’s whole period of marketing authorisation system divided into five periods according to changes in legislation (1965–1982, 1983–1987, 1988–1993, 1994–2004 and 2005–2021).

## Methods

The study aimed to assess how safety-related statutory actions, particularly product-specific safety information, were implemented for herbal products under the pharmaceutical legislations during the period of 1965–2021. The following special aspects were studied: (1) when herbal products got their marketing authorisations, registrations, and licenses [9], i.e., were approved to the market as herbal products in Finland; (2) how SmPCs, PLs and their predecessors of those herbal products in different legislative periods were implemented into practice and what was their appearance rate; and (3) what was the presence of contraindications (including special warnings and precautions) in the product information contents. This study defines a contraindication (including special warnings and precautions) as a specific situation where the product fulfilling the definition of herbal medicinal product or traditional herbal medicinal product as defined in Directive 2004/24/EC [2] must not or should not be used because it could harm the user. The five most common contraindications (including special warnings and precautions) up to 2012 were included in the study. Later, it was decided to use them until the end of this study. These five related to certain organs, e.g., cardiac conditions, liver or kidney functions, or they were, e.g., cause allergic or hypersensitivity reactions. Because the product-specific information is intended to ensure safe medicinal use, this information was selected for closer examination.

The material for this study consisted of approved product information documentation of all herbal products that had been granted a marketing authorisation, a registration, or a license and their product information documents under pharmaceutical supervision in Finland during 1965–2021 (Table 1). This material was collected in the paper and electronic format from the archives of the Finnish Medicines Agency Fimea. The study material contained all product labelling texts, package leaflets and their national predecessors called brochures, as well as the summaries of product characteristics (SmPCs) for herbal products that fulfilled the EU definitions of herbal medicinal product and traditional herbal medicinal product [2].

The study period 1965–2021 was divided into five periods according to the changes in pharmaceutical legislation (Table 1) [9]. The last five years 2017–2021 were reviewed also year by year. Detailed description of the legislative changes concerning herbal products under pharmaceutical legislation during 1965–2007 in Finland have been presented elsewhere [9]. Since 2007, no legislative changes regarding herbal medicinal products have been introduced into the EU legislation [10] nor to the national pharmaceutical legislation in Finland. Thus, the legislation remained the same during our most current study period of 2007–2021.

**Table 1:** Pharmaceutical legislation used as a reference for assessing implementation of product information of herbal products

	Medicinal product*	Medicine-like product*	Patient information material and special markings	Acts in Finnish pharmaceutical legislation and transitional provisions
2021 − 2005	Herbal medicinal product (HMP) and traditional herbal medicinal product (THMP) (2004-> in the EU Directive)	None	SmPC + PL + product labelling (as soon as MAs and renewals were granted) Phrase “TRADITIONAL HERBAL MEDICINAL PRODUCT” on package leaflets and product labellings [11]	Act 853/2005 [12] came into force on 7 Nov 2005 -fulfilling the definition for HMP to apply MA 31 Dec 2007 at the latest -fulfilling the definition for THMP, the old MA of HR was renewed as THMP
2004 - 1994	Medicinal product (1994-)	Herbal remedy (1994)	SmPC + PL + product labelling (as soon as authorisations were granted) Phrase ”HERBAL REMEDY” on product labellings [8]	Act 1046/1993 [13] came into force on 1 Jan 1994 -products under the previous Acts have been marked acc. to the new legislation -5 years scale (1995 to 1999) to apply an HR acc. to the original decision date; labelling to be updated 31.12.1995 at the latest
1995: Finland joined the European Union				
1994: Finland’s European Economic Area Agreement				
1993 − 1988	Medicinal product (1987-)	Section 68 subsections 1** and 3 (1987)	Brochure + product labelling ** Phrase ”MEDICINE-LIKE PRODUCT - EFFICACY NOT PROVEN AS REQUIRED FOR MEDICINES” on brochures and product labellings [14, 15]	Act 395/1987 [16] came into force on 1 Jan 1988 -products under the previous Acts were allowed to be on the market onwards
1987 − 1983	Medicinal product (1983-)	Sections 10e and 10f** (1983) + products under food legislation with medicine-like indication	Leaflet/brochure + product labelling (as soon as authorisations were granted) ** Phrase “THIS PRODUCT IS NOT CHECKED AS REGULATED FOR MEDICINES” on leaflets/brochures and product labellings [17]	Act 56/1983 [18] came into force on 1 Jul 1983** -to apply ** 31 Oct 1983 at the latest -> allowed to be on the market until the application has been solved
1982 − 1965	Pharmacy product (1964-)	None	Product labellings	Act 505/1963 [19] came into force on 1 Apr 1964 -to apply within four months -> allowed to be on the market until the final MA decision
1964: Modern marketing authorisation system was introduced in Finland [20].				

*The development of herbal product terms in the Finnish (and EU) pharmaceutical legislation(s) have been presented in detail in the reference [9]

### Data analysis

The study was carried out with a qualitative content analysis. The data were manually collected product by product using the list presented in Table 2. Each identified item of product-specific safety-related information in SmPCs, PLs, their predecessors and product labellings were documented by dividing them into separate Excel and Word sheets. The information items used as data in this study have been bolded in Table 2. Thus, this study focussed on studying implementation of the contents of the product information, not evolution of legislation.

**Table 2:** List of information collected from the study material. Bolded points discussed in this study

Does the product fulfill the definition of the herbal medicinal product or traditional herbal medicinal product?
Dates for granting, transition and ending of marketing authorisations, registrations, and licenses
SmPCs, PLs, brochures and product labellings (full texts saved for the analysis)
Compositions, pharmaceutical form, statements of ethanol, lactose, gluten, sucrose, sweetener, storage conditions, shelf life (unopen, after the first opening), distribution channel
Indication, posology, posology for children, notes for elderly, liver, kidney, notes for the onset of effect, the recommended duration of use
Side-effects, interactions with grapefruit juice, food, milk, heart medicine, **contraindications (including special warnings and precautions) for heart**,** kidney**,** liver**,** gastro-intestinal**,** allergy**, overdose, warnings regarding children, to drive or use machines, pregnancy and breastfeeding, the prohibition regarding children

## Results

### The herbal medicinal products and their predecessors under study period 1965–2021 in Finland

The final study material consisted of 207 herbal medicinal products after excluding six products because no data was found for them. The total numbers of granted, valid and expired marketing authorisations, registrations, and licenses for herbal medicinal products and their predecessors in Finland are presented in Table 3 according to five legislative sections. The numbers contain products authorised during the previous legislations and transfer from previous legislations to the subsequent legislations under transitional provisions. Some products have stayed on the market under several – or even each – periods of pharmaceutical legislations.

**Table 3:** Marketing authorisations, registrations, and licenses for HMPs and their predecessors according to legislative sections

	1964–1982	1983–1987	1988–1993	1994–2004	2005 - 2021	2017	2018	2019	2020	2021
Granted (n)	13	46	95	33	23	4	4	3	1	0
Valid (n)	13	57	147	179	105	30	32	32	32	30
Expired (n)	2	5	1	97	77	2	3	1	2	2

n = number of products

New medicine-like products were introduced for the first time in Finland in 1983 [18]. Products under section 10e were sold in pharmacies only. The products under section 10f were supervised under the Degree of the Ministry of Trade and Industry [21]. Although they were sold and marketed under the Board of Trade, their medicine-like indications were granted by the Finnish medicine authority. For this reason, 10f products were excluded from the total amount of marketing authorisations, registrations and licenses. Authorised herbal medicinal products remained as they were under the previous legislation.

In 1988, medicine-like products were transferred from the old legislation sections 10e and 10f to the new legislation Section 68, subsections 1 and 3 [16]. These products were allowed to remain on the market as they were prior to the changes in the pharmaceutical legislation [16]. No differences were introduced to authorised herbal medicinal products compared to other authorised medicines.

Many medicine-like products remained on the market as herbal remedies if the product labelling was updated by December 31, 1995, at the latest [13]. However, many marketing authorisation applications for herbal remedies were not submitted for the procedure before the end of the transitional provisions, and for this reason, these granted marketing authorisations ended.

Twenty-seven granted marketing authorisations for herbal remedies and herbal medicinal product-like medicinal products ended before December 31, 2007. This was the endpoint of a transition period in the legislation to submit new marketing authorisation applications for herbal medicinal products and to keep these products on the market until the assessments of new marketing authorisation applications were finalised. Those marketing authorisations for herbal remedies that did not fulfil the definition of a herbal medicinal product but fulfilled the definition of a traditional herbal medicinal product, and registration requirements, were changed via renewal procedures to traditional herbal medicinal products. These products were allowed to be on the market until the renewal reviews were completed.

In 2012, (seven years after the current pharmaceutical legislation came into force), the marketing authorisations for 19 herbal medicinal products and registrations for eight traditional herbal medicinal products were valid. The transitional period allowed those products under previous legislation to be on the market as long as their new applications were evaluated, and the number of valid authorisations remained quite high.

Marketing authorisations for 18 herbal medicinal products and registrations for 12 traditional herbal medicinal products were valid in the year 2021. Two marketing authorisations expired before the endpoint of this study. The number of valid authorisations decreased after the assessment work progressed during the long transitional period after the current legislation came into force. The situation remained more-or-less unchanged for the last five years.

Two herbal medicinal products for which marketing authorisations were granted for the first time in the first period (1964–1982) and one herbal medicinal product for which marketing authorisation was granted first time in the second period (1983–1987) as pharmacy products, had the valid marketing authorisations of herbal medicinal products at the end of the study period. These products are still on the market in Finland.

### Appearance of SmPCs, PLs and their predecessors for herbal products under pharmaceutical legislations

**Fig. 1:**
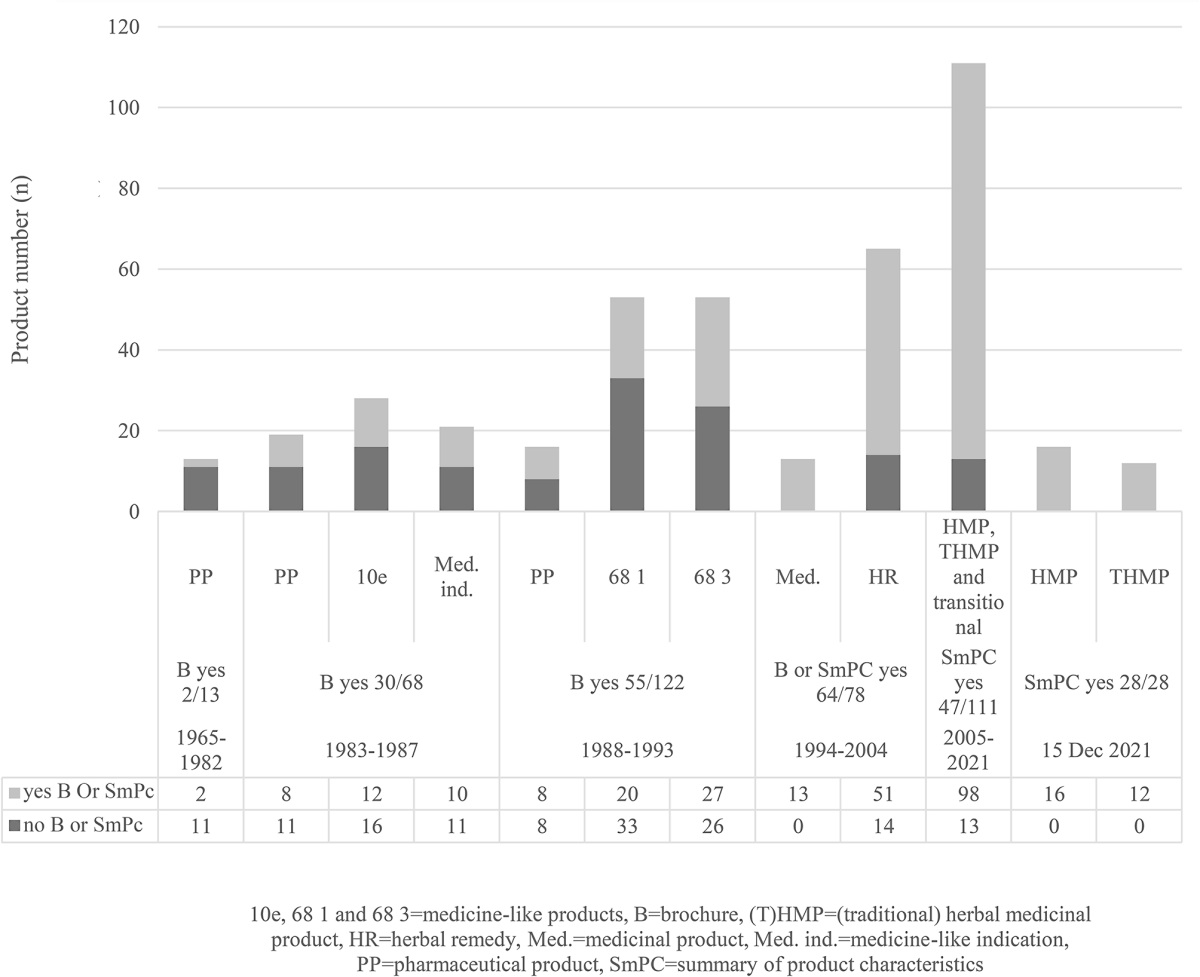
The appearance of brochures and SmPCs (including PLs) of HMPs and their predecessors in Finland

The appearance of SmPCs and brochures for herbal medicinal products and their predecessors in Finland are presented in Fig. 1. SmPC and PL were introduced into the Finnish legislation with the Medicines Act 1046/1993 [13]. Prior to this legislation, brochures were used for similar kinds of purposes than SmPCs. Pharmaceutical products, later medicinal products, were transferred between 1965 and 2021 from legislation to legislation without any reapplication procedure. A special group of products was the one with medicine-like indication granted by the Medicines Authority and supervised by the Board of Trade (1983–1987). Some of these products had a brochure and with the next change in the pharmaceutical legislation, these products transferred completely under the pharmaceutical legislation.

SmPCs and PLs were concurrently introduced into the national pharmaceutical legislation. In the first period (1964–1983), the brochure followed the product in very few cases (two products out of 13) (Fig. 1). The second period (1983–1987) brought brochures to many products under the pharmaceutical legislation (30 products out of 68). On the third period (1988–1993) number of products was higher, but more than half of the products were on the market without brochures (55 products out of 122 with a brochure).

The Finnish pharmaceutical legislation changed a lot after the European Economic Area (EEA) Agreement came into force in 1994 in Finland. SmPCs and PLs were included in the Finnish pharmaceutical legislation as part of the EU legislation. Herbal medicinal product-like medicinal products and herbal remedies in the fourth period (1994–2004) had SmPCs and PLs more often than were without them (64 out of 78). All herbal medicinal product-like medicinal products had SmPC and PL. Of the herbal remedies 78% had SmPC and PL.

After the EU Directive 2004/24/EC [2] came into force in Finland in 2005, and after assessment procedures were finalised, all herbal medicinal products and traditional herbal medicinal products have contained SmPCs and PLs (Fig. 1).

### **The appearance of five contraindications (including special warnings and precautions) in the product-specific information**

**Fig. 2:**
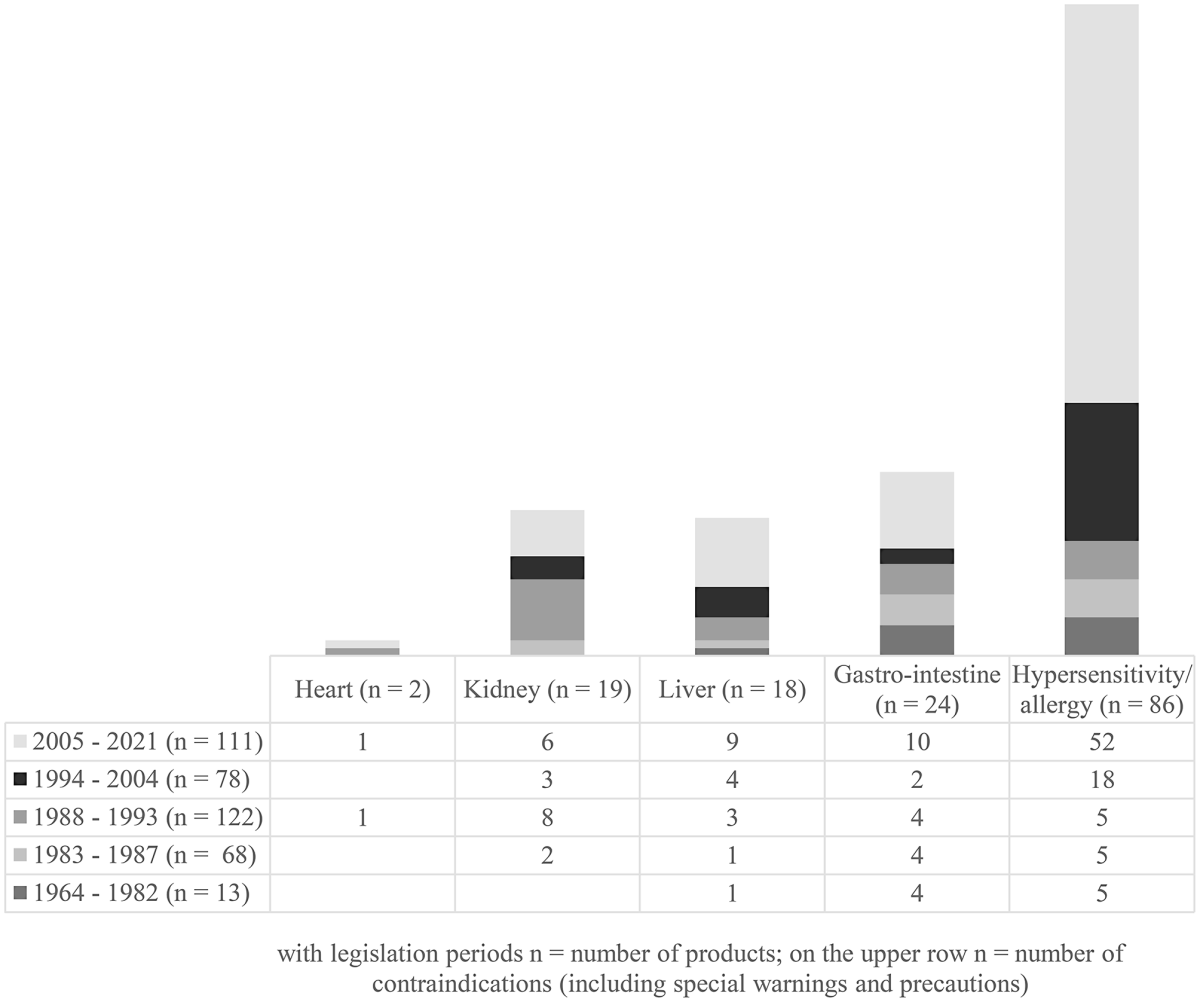
Contraindications (including special warnings and precautions) found in product information according to legislation periods

Contraindications (including special warnings and precautions) found in the study material are presented in Fig. 2. They concern heart, kidney, liver, gastro-intestine and hypersensitivity/allergy.

## Discussion

This unique study material covered the whole period of medicines’ marketing authorisation system started in 1964 in Finland. It provided the possibility to follow the implementation of statutory safety-related product information for herbal products during the study period 1965–2021. The results can be considered comprehensive, because the study material contained all approved product information documents found from the Fimea’s archives. Research material can also be considered reliable because it was fully based on the valid legal regulations.

Three main findings can be described. The first one relates the herbal medicinal products and their predecessors under study period. Several herbal products were transferred from one legislative section to another through transitional provisions. It took quite long time for the products to fulfil the valid new legislation. As requirements became stricter, product licenses and authorisations expired for one reason or another. These reasons were not investigated in more detail in this study. The second finding relates the appearance of SmPCs, PLs and their predecessors for herbal products under pharmaceutical legislations. After Finland became a member of the EU, SmPCs and PLs have gradually been included in herbal products. Currently, all (traditional) herbal medicinal products have included both SmPCs and PLs. The third finding relates to the appearance of five contraindications (including special warnings and precautions). Hypersensitivity/allergy was the most frequently included contraindication (including special warning and precaution) in the texts more often presented during the last two legislative sections. The background for this phenomenon was not studied but it might be related to general precautionary principle to give a special warning for user. Gastro-intestinal, kidney and liver contraindications (including special warnings and precautions) were found in some of the products, but more seldom and less indefinite way. These product safety information texts had increased during the last legislative section of 2005–2021.

Related to the topic of contraindications (including special warnings and precautions), the work of the European Medicines Agency (EMA) Committee on Herbal Medicinal Products (HMPC) has had an impact on the information content. HMPC was established in 2004 according to Directive 2004/24/EC, and it started preparations for EU herbal monographs and list entry texts [2]. Both EU herbal monographs and list entry texts serve as a harmonised base for SmPCs for (traditional) herbal medicinal products and offer harmonised, assessed information for better patient safety. This change has among other things improved the content of product safety information, such as contraindications, precautions, and special warnings. Finland has used EU monographs and community list entry texts from the beginning. The only exceptions have been those single EU herbal monographs Finland has presented divergent opinions. At the same time, the quality review of documents’ (QRD) templates, first time introduced in 1996, with the addendum [22, 23], readability testing [24], first time introduced in 1998, and adverse reaction system have improved the product information intended for patients. Until 2005, these QRD templates and readability guidelines were used for herbal remedies and their predecessors only nationally, whenever possible.

### Study limitations

As a limitation might be seen that this study contained a relatively small number of herbal products. Only 213 authorised, registered, or licensed herbal products in Finland were found in the Finnish marketing authorisation system between 1965 and 2021. Of these, six products were excluded from the study due to missing data and remaining 207 were included in this study. No specific selection was used to include or exclude herbal products. It also remained unclear whether some product information documents had been lost over the years, but all existing documentation was included in the study.

### Further research

Many additional safety-related research topics can be found in Table 2. One very interesting topic relates to children because of their vulnerability. Another interesting topic is how the active substances of these products have been written during the study period, and how well these names of active substances have been described for safe use of these products. Additionally, close look on general warnings, topics for granted indications, posologies and active substances that have remained or disappeared during the study period would be interesting research topics especially from a safety-related point of view.

## Conclusion

This study is based on a unique archive material covering years 1965–2021 in Finland. It gives information on how medicine-like products were first taken under the pharmaceutical legislation, and how the current situation in product safety information to consumers has gradually been reached. It shows successful national implementation of statutory product information for herbal products and how safety-related product information on contraindications (including special warnings and precautions) has improved during the study period, particularly since legislative changes in 2005. Safety-related information has, in one way or another, been part of the product-specific information required by national pharmaceutical legislation in Finland and in European Community legislation, and later in EU legislation. Currently all herbal products under pharmaceutical supervision are medicinal products which are controlled like any other medicinal products for patient safety.

## Data Availability

Data are available from the corresponding author on reasonable request.

## Abbreviations

B: Brochure
EC: European Community
EEA: European Economic Area
EEC: European Economic Community
EMEA/EMA: European Agency for the Evaluation of Medicinal Products (1995–2004) / European Medicines Agency (since 2005)
EU: European Union
FIMEA: Finnish Medicines Agency
HMP: Herbal medicinal product
HMPC: Committee on Herbal Medicinal Products
HR: herbal remedy
MA: marketing authorisation
PL: Package leaflet
PP: pharmaceutical product
QRD: Quality Review of Documents
SmPC: Summary of product characteristics
(T)HMP: (traditional) herbal medicinal product
